# A Community-Based Resistance Training Exercise for Post-Stroke Patients with Sarcopenia: Bridging Institutional and Community-Based Rehabilitation in a Multicenter, Randomized Controlled Trial

**DOI:** 10.3390/life15050748

**Published:** 2025-05-07

**Authors:** Dongheon Kang, Jiyoung Park

**Affiliations:** 1 Department of Healthcare and Public Health Research, National Rehabilitation Center, Ministry of Health and Welfare, Seoul 01022, Republic of Korea; luxpooh@gmail.com; 2 Department of Safety and Health, Wonkwang University, Iksan 54538, Republic of Korea

**Keywords:** stroke, sarcopenia, high-speed power training, community-based rehabilitation, randomized controlled trial

## Abstract

Sarcopenia is a prevalent and debilitating condition among stroke survivors, characterized by the progressive loss of muscle mass and function. This multicenter, randomized controlled trial aims to evaluate the effects of a community-based high-speed power training (HSPT) program tailored for individuals with post-stroke sarcopenia. The intervention bridges the gap between hospital-based rehabilitation and long-term community reintegration by offering physician-supervised, progressive resistance training sessions conducted twice weekly for eight weeks. Participants are assessed on muscle strength, physical performance, balance, body composition, and gait before and after the intervention. The study utilizes validated tools such as handgrip dynamometry, dual-energy X-ray absorptiometry (DXA), short physical performance battery (SPPB), and timed up and go (TUG) to comprehensively evaluate outcomes. Through stratified randomization and a double-blind design, the trial seeks to minimize bias and maximize clinical relevance. The results from this protocol are expected to inform evidence-based guidelines for stroke rehabilitation and support scalable community-based exercise programs aimed at improving functional recovery and quality of life in this population.

## 1. Introduction

Stroke is one of the leading causes of long-term disability and mortality worldwide, significantly impacting the quality of life for survivors. In South Korea alone, approximately 105,000 individuals experience their first or recurrent stroke annually, making stroke a major contributor to the national healthcare burden [1,2]. The recovery period immediately following a stroke is critical, as the central nervous system demonstrates heightened neuroplasticity, enabling substantial functional improvements through targeted interventions [3,4]. However, many stroke survivors face challenges in maintaining effective rehabilitation after hospital discharge, leading to a gap in long-term recovery and community reintegration [5]. In South Korea, many post-stroke patients lose access to rehabilitation programs after hospital discharge due to the limited availability of inpatient rehabilitation beds. This discontinuity may result in functional decline, increased dependency, and reduced community reintegration. According to national reports, community rehabilitation services remain underutilized despite high demand. Therefore, bridging institutional and community-based rehabilitation is critical to ensure continuous support for functional recovery.

Sarcopenia, characterized by a progressive decline in skeletal muscle mass and strength, is prevalent among stroke survivors, with reported rates ranging from 14% to 54% depending on diagnostic criteria and population characteristics [6,7]. Post-stroke sarcopenia presents unique challenges, including rapid muscle atrophy, fiber type alterations, and neuromuscular impairments caused by factors such as disuse, malnutrition, and spasticity [8,9]. These changes affect both paretic and non-paretic limbs, resulting in bilateral functional decline [10]. Addressing sarcopenia is essential for improving mobility, reducing secondary complications, and facilitating successful community reintegration following hospital-based rehabilitation [11,12].

Resistance training is a well-established intervention for mitigating sarcopenia, enhancing muscle strength, mass, and functional performance [13]. Traditional resistance training primarily focuses on slow, controlled movements to strengthen muscle. However, high-speed power training (HSPT) differs significantly in prioritizing the rapid generation of force, which is critical for functional movements such as walking, sit-to-stand transitions, and balance recovery [14]. HSPT specifically targets fast-twitch muscle fibers, which are disproportionately affected in stroke survivors, helping to counteract muscle atrophy and improve reaction time [14,15]. Unlike conventional resistance training, which primarily enhances maximal strength, HSPT improves both strength and explosive power, making it particularly effective in reducing fall risk and enhancing functional independence [14]. Despite its potential, evidence on the application of HSPT specifically for stroke survivors with sarcopenia remains limited.

Community-based interventions are essential for bridging the gap between hospital discharge and long-term rehabilitation. Before participating in such programs, stroke survivors are required to obtain a physician’s referral. Physicians assess the patient’s medical condition, evaluate potential risks associated with exercise, and provide guidance on precautions for community-based exercise programs. This information is incorporated into a referral letter that serves as fitness professionals’ basis for exercise program planning [16,17]. Exercise specialists utilize the physician’s recommendations to design and implement tailored programs that ensure safety and efficacy. This approach minimizes adverse events and optimizes rehabilitation outcomes, particularly for individuals with complex conditions such as post-stroke sarcopenia [15,18]. While endurance-based interventions have been studied, they may not adequately address the rapid type II muscle fiber loss and impaired power output seen in post-stroke sarcopenia. High-speed power training (HSPT) has demonstrated superior outcomes in improving neuromuscular responsiveness, sit-to-stand performance, and fall prevention among older adults and stroke survivors [19,20,21,22,23]. These findings provide a rationale for evaluating HSPT as a specialized approach for mitigating sarcopenia in community settings.

Despite numerous RCTs evaluating resistance training for stroke survivors, most have employed traditional slow-velocity modalities that primarily target maximal strength. These studies often report modest improvements in physical function but lack emphasis on power generation, a key component for mobility and fall prevention [20,21]. Moreover, few trials have adopted community-based frameworks with structured medical oversight. Our protocol addresses these gaps by implementing high-speed power training (HSPT), which has shown superior benefits in older adults and stroke populations in improving explosive strength and dynamic function [22,23]. This study is among the first to evaluate a community-integrated HSPT program tailored for post-stroke sarcopenia, with rigorous outcome assessments and multicenter generalizability.

This study aims to develop and evaluate physician-supervised, community-based high-speed power training for stroke survivors with sarcopenia through a multicenter, randomized controlled trial. By bridging the gap between hospital-based rehabilitation and community life, the protocol offers a structured, accessible, evidence-based intervention that integrates medical oversight. The findings from this study will contribute to the growing body of evidence on sarcopenia management and inform the development of standardized post-stroke rehabilitation strategies, ultimately improving functional outcomes and quality of life for stroke survivors.

## 2. Materials and Methods

### 2.1. Participants

This study will enroll 120 participants, with 30 recruited from each of the four participating institutions. Participants will be recruited from rehabilitation centers, hospitals, and community-based rehabilitation programs. Eligible participants must be adults aged 50 years or older who have been diagnosed with stroke at least six months prior to enrollment. While no upper limit is imposed, the range of post-stroke durations (e.g., 6 months to several years) will be accounted for through stratified randomization via baseline functional status. Participants must be medically stable and cleared by a physician to participate in an exercise program. Additionally, participants must meet the diagnostic criteria for sarcopenia as defined by the European Working Group on Sarcopenia in Older People 2 (EWGSOP2) [24]. According to EWGSOP2, sarcopenia is diagnosed based on the presence of low muscle strength, low muscle mass, and/or poor physical performance, with low muscle strength being the primary determinant.

Muscle strength will be assessed as the primary diagnostic criterion for sarcopenia. Handgrip strength will be measured using a dynamometer, with cut-off values of less than 27 kg for men and 16 kg for women indicating low muscle strength. Alternatively, the five-times sit-to-stand test, which evaluates lower limb strength and functional ability, will be used as an additional criterion, with a time of 15 s or more suggesting muscle weakness.

Muscle mass will be evaluated using bioelectrical impedance analysis (BIA) or dual-energy X-ray absorptiometry (DXA). The appendicular skeletal muscle mass index (SMI) will be calculated by dividing the muscle mass of the upper and lower limbs by height squared (kg/m^2^). According to EWGSOP2 guidelines, a low muscle mass will be defined as an SMI of less than 7.0 kg/m^2^ for men and less than 5.5 kg/m^2^ for women.

Physical performance will be assessed to determine the severity of sarcopenia. Gait speed will be measured using a 4 m walk test, with a walking speed of 0.8 m/s or lower considered indicative of poor physical performance. In addition, the short physical performance battery (SPPB) score will be used as a composite measure of functional performance, including balance, gait speed, and chair stand tests. A total SPPB score of 8 or lower will be considered evidence of poor physical performance and increased sarcopenia severity.

Participants must also be able to walk with or without assistive devices such as a cane or walker.

Exclusion criteria include severe medical conditions that may hinder safe participation, such as uncontrolled cardiovascular disease, unstable angina, or a recent myocardial infarction. Participants with severe musculoskeletal disorders, progressive neurodegenerative diseases (e.g., Parkinson’s disease or amyotrophic lateral sclerosis (ALS)), or uncontrolled diabetes with neuropathy affecting mobility will also be excluded. Cognitive impairment, defined as a Mini-Mental State Examination (MMSE) score < 20, will serve as an exclusion criterion due to its potential impact on adherence and safety. Furthermore, individuals who have engaged in a structured resistance training program within the past three months or have contraindications for exercise, such as severe uncontrolled hypertension (systolic blood pressure ≥ 180 mmHg or diastolic blood pressure ≥ 110 mmHg) or severe osteoporosis with a history of fragility fractures, will not be eligible for participation.

### 2.2. Study Design

This study will be conducted across four medical and rehabilitation centers and follow a randomized, double-blind, parallel-group design to assess the impact of a community-based resistance training program for stroke survivors with sarcopenia. A total of 120 participants will be randomly assigned in a 1:1 ratio to either the intervention group, which will engage in structured resistance training, or the control group, which will continue their routine activities without a structured exercise program. To ensure unbiased results, assessors conducting evaluations will remain blinded to group assignments throughout the study.

The study will be conducted at (1) the National Rehabilitation Center, (2) Bucheon SM Hospital, (3) Korea University Anam Hospital, and (4) the National Health Insurance Service Ilsan Hospital. Participants will be recruited from outpatient rehabilitation programs at these institutions. Eligibility will be confirmed through a pre-screening process, followed by an informed consent procedure to ensure that participants fully understand the study objectives and methodology.

Once enrolled, participants will undergo baseline assessments before randomization. The intervention group will participate in a high-speed power training program designed to enhance muscle strength, functional performance, and mobility. The program will be conducted twice a week for 8 weeks, with each session lasting 60 min and incorporating progressive resistance exercises. Meanwhile, the control group will maintain their regular daily activities without any structured exercise intervention while continuing to receive standard medical care.

**Figure 1:**
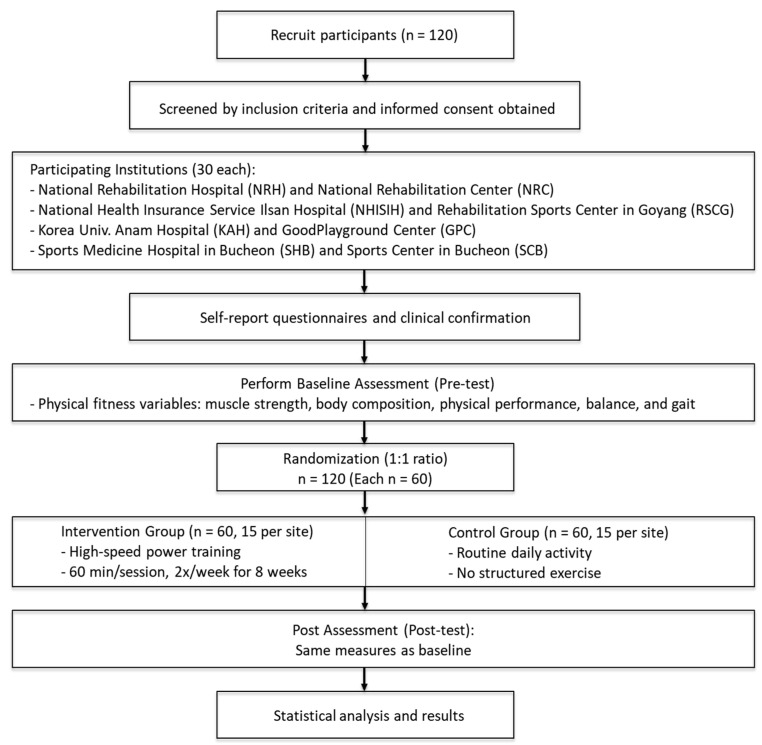
Study flowchart.

Outcome assessments will be conducted at two key time points: before the intervention (baseline) and after completing the 8-week program (post-intervention). The overall study procedure, including recruitment, group allocation, intervention, and assessments, is illustrated in Figure 1. The outcome measures will include muscle strength, physical function, body composition, and cardiopulmonary fitness. The collected data will undergo statistical analysis to evaluate the effectiveness of the resistance training program in mitigating sarcopenia-related functional decline among stroke survivors.

This study follows the SPIRIT guidelines [25] and has been approved by the Institutional Review Board (IRB) of the National Rehabilitation Center (NRC IRB No. 050432021). The study protocol has been registered under Study Registration Number KCT0007521. The findings are expected to provide valuable insights into the role of community-based resistance training in stroke rehabilitation and its potential to enhance post-rehabilitation health outcomes.

### 2.3. Randomization

This study employs a computer-generated, stratified block randomization method to ensure a balanced allocation of participants across study groups. After completing baseline assessments, eligible participants will be randomly assigned in a 1:1 ratio to either the intervention group (high-speed power training program) or the control group (standard care). To maintain allocation concealment, the randomization process will be conducted independently by a statistician who is not involved in participant recruitment, assessment, or intervention delivery.

Randomization will be stratified by study site and baseline functional status to ensure that both groups have comparable distributions of key characteristics. To achieve this, four to six block sizes will be used, and assignments will be sealed in opaque, consecutively numbered envelopes prepared by an independent research assistant. These envelopes will be opened only after the participant is enrolled and their baseline assessment is completed.

To minimize bias, this study follows a double-blind design, where participants and outcome assessors remain unaware of group assignments throughout the study period. Only the exercise specialists administering the intervention will have access to group allocation. Participants will be instructed not to disclose their group assignment to assessors during follow-up evaluations.

This randomization strategy ensures that the study groups remain well balanced, reducing the risk of selection bias and enhancing the reliability of the study findings.

### 2.4. Randomization and Blinding

This study employs a stratified block randomization method to ensure a balanced allocation of participants across the intervention and control groups. Randomization will be stratified by study site and baseline functional status, with block sizes of four to six, to minimize imbalance between groups. An independent statistician who is not involved in participant recruitment, assessment, or intervention delivery will generate the random allocation sequence using computerized randomization software. Group assignments will be sealed in opaque, consecutively numbered envelopes, which will only be opened after participant enrollment and baseline assessment.

To maintain blinding, this study follows a double-blind design, where both participants and outcome assessors will remain unaware of group assignments throughout the study. Only the exercise specialists delivering the intervention will have access to group allocation. Participants will be instructed not to disclose their group assignment during follow-up evaluations to prevent bias.

Additionally, data analysts will be blinded to group assignments during statistical analysis to ensure the unbiased interpretation of results. This randomization and blinding protocol is designed to reduce selection bias, measurement bias, and potential confounding factors, ultimately enhancing the validity and reliability of the study findings.

### 2.5. Protocol

This study follows a standardized protocol to ensure consistency in participant recruitment, intervention delivery, and outcome assessment across multiple study sites. The study protocol consists of four key phases: screening and enrollment, baseline assessment, intervention, and post-intervention assessment.

Participants will be recruited from outpatient rehabilitation programs at participating institutions. Eligibility will be determined through a pre-screening process based on inclusion and exclusion criteria. Those who meet the eligibility criteria will proceed to the informed consent process, where they will receive detailed explanations regarding the study’s objectives, procedures, potential risks, and expected benefits.

After enrollment, all participants will undergo a comprehensive baseline assessment, which includes collecting demographic and clinical data such as age, sex, stroke history, and comorbidities. Functional assessments will be conducted to evaluate muscle strength, physical performance, body composition, and cardiopulmonary fitness. These baseline evaluations will establish pre-intervention values for subsequent comparisons.

Following randomization, the intervention group will participate in an 8-week high-speed power training (HSPT) program conducted twice per week, with each session lasting 60 min. The exercise protocol will include a 10 min warm-up, a 40 min main training session focused on progressive resistance exercises emphasizing power development, and a 10 min cool-down incorporating stretching and relaxation techniques. In contrast, the control group will continue their daily routine without participating in structured resistance training while still receiving standard medical care.

Upon completing the 8-week program, all participants will undergo a post-intervention assessment identical to the baseline evaluation. This assessment will measure changes in muscle strength, gait speed, balance, body composition, and cardiopulmonary fitness to determine the effects of the intervention.

This protocol ensures a structured and reproducible methodology for intervention implementation and data collection, thereby enhancing the reliability and validity of study findings across multiple sites.

### 2.6. Intervention Program

The intervention program is based on the principles of HSPT and aims to improve muscle strength, power, and functional mobility in stroke survivors with sarcopenia. Structured training is conducted twice per week for 8 weeks, with each session lasting 60 min and consisting of three sequential phases: warm-up, main exercise, and cool-down. The detailed composition of the program is presented in Table 1.

The warm-up phase (10 min) includes aerobic walking and static or dynamic stretching to increase body temperature, enhance circulation, and prepare joints and muscles for high-velocity movements.

The main exercise phase (40 min) consists of resistance training targeting the lower body, trunk, and upper body, using TheraBand (Hygenic Corporation, Akron, OH, USA) as the primary resistance tool. Exercises are performed according to a high-speed power training protocol characterized by an explosive concentric contraction, followed by a brief 1 s pause, and then a controlled eccentric contraction lasting more than 2 s. This sequence is designed to activate fast-twitch muscle fibers, which are often compromised in stroke survivors, and to improve rapid force production essential for functional tasks such as gait, sit-to-stand transfers, and balance recovery.

The exercise selection includes squats, lunges, and bridges for the lower body; leg raises, reverse crunches, sit-ups, back extensions, and deadlifts for the trunk; and chest presses, back rows, shoulder presses, biceps curls, and triceps extensions for the upper body. The intensity and resistance are progressively increased based on individual capability, with modifications made in accordance with the principles of progressive overload and task specificity. Exercise specialists closely monitor performance to ensure proper technique and safety throughout the program.

The cool-down phase (5 min) consists of light walking and flexibility exercises, aimed at promoting active recovery, reducing muscle soreness, and improving joint range of motion.

This training program is standardized across all participating study sites and delivered by experienced exercise professionals specializing in stroke rehabilitation. The protocol is individualized according to each participant’s physical condition and recovery status, integrating principles of safety, adaptability, and progression. By combining resistance training with speed-focused contraction patterns, the program is expected to enhance functional outcomes and support the community reintegration of stroke survivors with sarcopenia.

### 2.7. Outcome Measures

All participants will complete a comprehensive evaluation twice during the study: once prior to the intervention (baseline) and once again after the completion of 16 training sessions (post-intervention). Evaluations will be conducted by trained assessors who are blinded to group assignments. In addition to clinical and demographic characteristics, a range of physical health and function indicators will be measured to assess the effects of the intervention. These characteristics are summarized in Table 2.

#### 2.7.1. Muscle Strength

Muscle strength will be assessed using both grip strength and isokinetic muscle strength testing [26,27]. Grip strength will be measured using a hand dynamometer (TKK-5401; Takei Scientific Instruments, Tokyo, Japan), which provides a reliable index of general upper limb strength. It is also widely used as a proxy for overall muscle function and sarcopenia screening. For the lower limbs, isokinetic testing will be conducted using the Humac Norm system (CSMi, Stoughton, MA, USA) to quantify peak torque and muscle endurance at preset angular velocities. This method allows for the objective evaluation of dynamic strength in the major muscle groups, particularly in the knee extensors and flexors, which are critical for postural control and mobility in stroke survivors.

#### 2.7.2. Body Composition

Body composition will be evaluated using dual-energy X-ray absorptiometry (DXA) with Discovery Wi (Hologic, Waltham, MA, USA) and bioelectrical impedance analysis (BIA) with InBody S10 (InBody, Seoul, Republic of Korea). DXA will be used to provide accurate measurements of whole-body lean mass, appendicular skeletal muscle mass, and body fat percentage. In addition, BIA will be employed to assess segmental lean mass and fat distribution. From these data, the skeletal muscle mass index (SMI) will be calculated as appendicular lean mass divided by height squared (kg/m^2^), which is a key diagnostic criterion for sarcopenia. These complementary methods offer a comprehensive view of changes in body composition resulting from the training program.

#### 2.7.3. Physical Performance

To assess physical performance, the short physical performance battery (SPPB) will be administered. This tool includes a timed gait speed test, repeated chair stands, and static balance tasks, collectively providing an index of lower extremity function. The SPPB is a validated instrument commonly used in geriatric and rehabilitation populations to quantify mobility and functional impairment [28].

#### 2.7.4. Balance and Gait

Both static and dynamic balance, as well as gait function, will be evaluated. The Berg Balance Scale (BBS) will be used to measure static balance across 14 functional tasks, with higher scores indicating better balance ability [29]. To assess dynamic balance and mobility, the timed up and go (TUG) test will be administered [30]. This test measures the time it takes for a participant to rise from a chair, walk three meters, turn around, walk back, and sit down again. In addition, the 10 m walk test (10MWT) will be used to assess usual gait speed over a short distance, an important predictor of community ambulation potential and functional independence in stroke survivors [31].

### 2.8. Participant Timeline

All participants will follow a structured timeline that includes screening, the baseline assessment, the intervention phase, and the post-intervention assessment. Initially, eligible participants will be recruited from outpatient rehabilitation centers at four participating institutions. A preliminary screening process will be conducted to confirm eligibility based on the study’s inclusion and exclusion criteria, and written informed consent will be obtained prior to enrollment.

Following consent, participants will complete a baseline evaluation prior to randomization. This assessment will include the collection of demographic and clinical information, as well as a comprehensive set of physical function measures such as muscle strength, body composition, physical performance, and balance. The assessment tools used for each domain are detailed in Table 2 and Table 3.

Following randomization, 120 participants will be allocated to either the intervention or control group. As previously detailed, the intervention group will complete an 8-week high-speed power training program, while the control group will continue their usual activities with general health education. This section serves to summarize the overall study timeline, consolidating key steps such as recruitment, baseline assessment, intervention, and post-intervention evaluation. This repeated measurement design will enable pre–post-comparisons to evaluate the effects of the training program on physical function and body composition in stroke survivors with sarcopenia. All procedures will be standardized across sites to ensure consistency in delivery and data collection, and the study timeline will be structured in accordance with the SPIRIT guidelines [25].

### 2.9. Statistical Analysis

The statistical analysis for this study will be conducted using IBM SPSS Statistics (version 26.0; IBM Corp., Armonk, NY, USA). Before the trial, a sample size calculation was performed using G*Power (version 3.1.2), assuming an effect size of 0.5, a significance level (α) of 0.05, and a statistical power of 80% (1–β). The effect size (Cohen’s d = 0.5) was selected based on prior studies showing the moderate-to-large effects of high-velocity or multicomponent resistance training in stroke or sarcopenic populations [19,20]. An anticipated attrition rate of 20% was assumed, consistent with previous 8–12-week intervention studies in community-dwelling stroke survivors [21], resulting in an adjusted target of 120 participants. Based on these assumptions and accounting for possible attrition, a total of 120 participants will be recruited—30 individuals from each of the four participating institutions.

Descriptive statistics will be computed to characterize the participants and evaluate baseline equivalence between the intervention and control groups. Categorical data will be presented as frequencies and percentages, while continuous data will be summarized as means with standard deviations or medians with interquartile ranges, depending on the distribution. To assess baseline group differences, independent *t*-tests or Mann–Whitney U tests will be used for continuous variables, and chi-square tests or Fisher’s exact tests will be applied for categorical variables.

The analysis will adhere to an intention-to-treat (ITT) principle. The normality of the outcome variables will be evaluated using the Shapiro–Wilk test. Depending on the distribution, either parametric or non-parametric tests will be selected accordingly. To examine intervention effects, two-way repeated-measures ANOVA will be employed with group (intervention vs. control) as the between-subject factor and time (pre- and post-intervention) as the within-subject factor. Interaction effects will be interpreted to determine the differential change over time between groups. Where sphericity assumptions are violated, appropriate corrections (e.g., Greenhouse–Geisser adjustment) will be applied.

All hypothesis testing will use a two-sided α level of 0.05. Estimates of intervention effect will be accompanied by 95% confidence intervals.

## 3. Discussion

This multicenter randomized controlled trial aims to assess the effectiveness of a community-based HSPT program for stroke survivors with sarcopenia. While resistance training has been widely recommended as a therapeutic strategy for sarcopenia, limited evidence exists regarding the use of high-velocity training specifically designed to restore functional capacity in post-stroke populations. This study addresses this gap by delivering a power-oriented resistance program in real-world community settings, which may enhance the continuity of care from hospital-based rehabilitation to independent living [19,20].

Stroke-related sarcopenia is distinct in its manifestation, often involving asymmetrical muscle wasting, impaired neuromuscular activation, and physical inactivity [21]. These impairments affect both paretic and non-paretic limbs and contribute to significant mobility limitations. High-speed power training prioritizes rapid force production and is particularly effective for targeting type II muscle fibers—fibers known to atrophy rapidly after stroke and aging. Previous studies have shown that such training improves gait, sit-to-stand performance, and overall balance more effectively than traditional slow-velocity strength training [22,23].

Importantly, this trial incorporates a community-based delivery model across four outpatient rehabilitation centers, ensuring external validity and real-world applicability. The decentralized structure allows the intervention to be embedded in routine rehabilitation practices, improving feasibility and access for stroke survivors. Furthermore, the standardized training protocol and supervision by qualified exercise professionals contribute to the safety and reproducibility of the intervention [32,33].

A notable strength of this study is its comprehensive outcome framework, including objective measures of muscle strength, body composition, physical performance, and balance. These multidimensional assessments are essential for evaluating the multifaceted impact of sarcopenia and its treatment [34,35]. The trial ensures methodological rigor and minimizes measurement bias by employing validated instruments and consistent procedures across all study sites.

However, certain limitations should be acknowledged. The relatively short intervention duration and lack of long-term follow-up are limitations. These factors may affect the generalizability of the findings and limit our ability to assess sustainability. The intervention period is limited to 8 weeks, and longer-term outcomes remain unexplored. Additionally, due to the nature of the intervention, blinding participants is not feasible, which may introduce performance bias. Lastly, heterogeneity in baseline functional status and cognitive ability among stroke survivors may influence intervention adherence and outcomes, despite efforts to standardize implementation. The sustainability of community-based rehabilitation programs depends on the competency and availability of community healthcare workers. In our model, trained exercise specialists deliver the intervention with supervision from physical medicine and rehabilitation (PM&R) physicians. Equipment requirements are minimal and adaptable to local settings. In implementing community-based programs, practical factors such as equipment availability, exercise space, and sustainability must be considered. Future studies should also evaluate the long-term impact of HSPT on sarcopenia and mobility in real-world settings. Stroke survivors with sarcopenia often have reduced exercise tolerance and require greater supervision. Ongoing monitoring by trained community health professionals may improve adherence and maximize intervention outcomes. Future implementation should consider strategies for regular supervision, continued training, and system integration into public health frameworks. Supportive policies, dedicated funding, and infrastructure are also essential to ensure the sustainability and scalable implementation of these programs across communities. Nevertheless, this study is expected to contribute valuable evidence regarding the role of HSPT in stroke rehabilitation, particularly in managing post-stroke sarcopenia. Its findings may inform clinical practice and guide the future development of community-integrated rehabilitation models for improving functional recovery and long-term health in this population.

## 4. Conclusions

This multicenter randomized controlled trial is designed to investigate the effects of a community-based high-speed power training (HSPT) program for stroke survivors with sarcopenia. By targeting neuromuscular deficits specific to post-stroke sarcopenia and implementing a structured, progressive intervention in real-world settings, this study aims to bridge the gap between hospital-based rehabilitation and long-term functional independence.

The proposed program incorporates principles of power training known to enhance muscle strength, mobility, and balance, which are critical for reducing fall risk and promoting community reintegration in stroke populations. Moreover, the inclusion of objective and multidimensional outcome measures strengthens the methodological rigor and clinical relevance of the findings.

This trial is expected to contribute valuable evidence supporting the feasibility and effectiveness of HSPT in community settings. The results may inform rehabilitation protocols and public health strategies for improving long-term outcomes among stroke survivors with sarcopenia. A key limitation of this study is the relatively short duration of the intervention (8 weeks), which may be insufficient to observe sustained functional gains or long-term adherence. Additionally, the absence of follow-up assessments limits our ability to evaluate the durability of treatment effects. Future studies should incorporate extended follow-up periods (e.g., 6–12 months) to assess the maintenance of functional improvements and long-term community reintegration. Ultimately, this study seeks to promote a sustainable model of community-based rehabilitation that addresses the persistent functional limitations and health risks faced by this vulnerable population.

## Figures and Tables

**Table 1:** Structure of the program.

Category		Contents
Warm-Up Cool-Down	Aerobic	Walking
	Flexibility	Static/Dynamic Stretching
Main Exercise Resistance Training	Lower Extremities	Squat, Lunge, Bridge
	Trunk	Deadlift, Leg Raise, Russian Twist, Crunch, Reverse Crunch, Side Crunch, Sit-up, Back Extension, Superman Position
	Upper Extremities	Shoulder Press, Biceps Curl, Triceps Extension, Chest Press, Back Row

**Table 2:** Baseline Clinical Profile and Physical Measurements of Participants.

Category	Variables	Units/Response Options
Clinical Profile	Chronological age	Age in years
	Biological sex	Sex (M/F)
	Classification of stroke	Stroke subtype
	Presence of comorbidities: hypertension, anemia, respiratory disorders, orthostatic hypotension, diabetes, cardiovascular disease, coronary interventions, epilepsy, psychiatric medications (e.g., antidepressants), recent lower back pain (within 4 weeks), joint pain affecting mobility, osteoporosis, and fracture history (spine, hip, or femur due to falls or bone fragility)	Confirmed or not
Physical Measurements	Systolic and diastolic	mmHg
	Stature	Height in centimeters
	Body weight	Weight in kilograms
	Body mass index	BMI category
	Days since hospital discharge	Elapsed time in days

**Table 3:** Outcome domains and assessment tools.

Outcome Domain	Assessment Tool
Muscle Strength	Handgrip Strength (Hand Dynamometer) Isokinetic Strength Test
Body Composition	Dual-Energy X-Ray Absorptiometry (DXA) Bioelectrical Impedance Analysis (BIA)
Physical Performance	Short Physical Performance Battery (SPPB)
Static Balance	Berg Balance Scale (BBS)
Dynamic Balance and Gait	Timed Up and Go Test (TUG) 10-Meter Walk Test (10MWT)

## Data Availability

The data will be made available by the authors upon reasonable request.
